# Novel Numerical Method for Studying Water Freezing on Surfaces Texturized by Laser

**DOI:** 10.3390/ma17246155

**Published:** 2024-12-17

**Authors:** Samih Haj Ibrahim, Tomasz Wejrzanowski, Christian W. Karl, Espen Sagvolden, Jakub Karwaszewski, Monika Pilz, Bartłomiej Przybyszewski, Rafał Kozera

**Affiliations:** 1 Technology Partners Foundation, Bitwy Warszawskiej 1920 r. 7A, 02-366 Warsaw, Poland; tomasz.wejrzanowski@technologypartners.pl (T.W.); bartlomiej.przybyszewski.dokt@pw.edu.pl (B.P.); rafal.kozera@technologypartners.pl (R.K.); 2 Department of Functional Materials Engineering, Faculty of Electronics, Telecommunications and Informatics, Gdańsk University of Technology, Narutowicza 11/12, 80-233 Gdańsk, Poland; 3 Faculty of Materials Science and Engineering, Warsaw University of Technology, Woloska 141, 02-507 Warsaw, Poland; 4 Polymer and Composite Materials, SINTEF Industry, Forskningsveien 1, 0373 Oslo, Norway; christian.karl@sintef.no; 5 Materials Physics, SINTEF Industry, Forskningsveien 1, 0373 Oslo, Norway; espen.sagvolden@sintef.no; 6 Process Chemistry and Functional Materials, SINTEF Industry, Forskningsveien 1, 0373 Oslo, Norway; monika.pilz@sintef.no

**Keywords:** CFD, freezing delay, laser texturization, icephobic surfaces

## Abstract

Within this study, a methodology for the numerical simulation of droplet freezing, including a micrometer texturized pattern, was developed. The finite volume method was then applied to simulate the behavior of water droplets. The procedure was divided into two processes: stabilization and freezing. In the stabilization step, the droplet was dropped onto the material surface and took an equilibrium shape. In the second step, additional energy equation and temperature boundary conditions were applied to perform freezing simulation. Based on the laser-texturized samples of polyurethane-coated metal substrates studied with freezing delay experiments, numerical models were generated, and droplet freezing simulations were performed. Three cases were studied—non-texturized and texturized with respectively linear and triangular patterns. The obtained simulation results of freezing time were compared with experimental measurements to evaluate the proposed methodology. The study revealed that despite the inability to predict accurate freezing delay time, the proposed methodology can be used to compare the freezing delay capabilities for different texturized patterns. Additionally, the proposed model renders it possible to analyze additional aspects of wetting and freezing of the droplet on rough surfaces, which may be helpful in understanding these processes.

## 1. Introduction

The hydrophobic effect of any substrate surface has attracted much wider research interest due to the increasing number of applications. Self-cleaning surfaces, rain-repellant windows, anti-biofouling ship hulls, corrosion-resistant coatings, and anti-icing surfaces are only a selection of potential applications [1,2,3,4,5]. Icephobicity, originating partially from the hydrophobic properties of surfaces, is also an important area of research connecting heat and mass transfer with phase change [6,7]. Studies on the icephobic properties of materials can be applied in many industries and applications, such as aerospace, wind turbines, power transmission, sea transportation and others [8]. Harsh cold-weather conditions can lead to icing, which can cause serious safety, economic and operational problems [9], such as ice accretion on aircraft surfaces leading to higher fuel consumption with additional risk to flight safety [10]. Wetting on rough surfaces can be observed in two wetting states. In the first case (Wenzel state), the liquid completely penetrates the roughness grooves (homogeneous wetting), and in the second case (Cassie–Baxter state), the liquid does not penetrate the rough surface due to the inclusion of air (heterogeneous wetting). The resulting contact angle is larger than in the case of the Wenzel state because the interface between the two substances is smaller [11,12]. The air trapped in the voids and stabilizing the Cassie–Baxter state leads to superhydrophobicity [13,14]. Superhydrophobicity with the Cassie–Baxter wetting state is commonly associated with icephobicity [15,16,17]. To obtain a superhydrophobic surface, a variety of methods can be used. Texturization is one of them, based on a change in the topography by introducing micro- and nanoscale patterns, often with a combination of chemical treatments in bulk materials [18,19,20], which renders it possible to obtain low surface energy favoring icephobic properties [21]. Concerning rough and chemically uniform surfaces, the droplet needs to be sufficiently large in comparison with the chemical heterogeneity or the scale of roughness as discussed in the literature [22,23]. As a prerequisite for measuring the contact angle according to Marmur, a ratio between the droplet diameter and the lateral extent of the roughness structures of at least three orders of magnitude is required [24].

Nanoscale roughness, compared to microscale, has been reported to exhibit greater influence on icephobic properties [25,26,27,28,29]. Additionally, air pockets present in the fine details of surface roughness below the water droplet facilitate enhanced water-repelling capability. However, the texture of superhydrophobic surfaces in which frost forms can lead to increased ice adhesion [30]. Nevertheless, the findings of several studies indicate that the correlation between water wettability and ice adhesion are inconclusive. The most widely accepted theory is that superhydrophobic surfaces with high water repellency exhibit improved anti-icing properties. Due to their effectiveness in delaying and/or reducing ice accumulation, snow and frost, these surfaces are reported as coatings with high icephobic potential [31,32,33,34,35,36]. Among those publications, several authors indicated the effect of contact angle hysteresis on ice adhesion strength [18,31,32,33,36], while others have reported reduced ice adhesion for high contact angle values coatings [31,32,33,34,35]. In contrast to those findings, many authors indicate no correlation or even a negative influence of superhydrophobic properties on icephobic behavior. For example, Chen et al. [37] suggested that superhydrophobic properties cannot decrease the ice adhesion. A similar relationship was partially found by Kulinich et al. [31] for rough hydrophobic surfaces with the exception that wetting hysteresis was well correlated with ice adhesion Meanwhile, Meuler et al. found a strong correlation for silicon-based materials, emphasizing that it is more pronounced for rigid materials than for elastomeric substrates [25].

Droplet freezing simulations on flat surfaces without the introduction of surface roughness were performed previously [3]. In the study mentioned, a numerical method was developed to analyze the anti-icing mechanism of the superhydrophobic surface. The Coupled Level Set-Volume of Fluid (CLVOF) and solidification-melting models were utilized to create a three-dimensional Computational Fluid Dynamics (CFD) model, which enabled tracking the morphological changes of droplets as well as simulating the phase transition process. The CLVOF method provides more fidelity in interface simulations than the regular volume of fluid method due to the additional level set function for continuous interface tracking [38]. Droplet freezing on surfaces with different contact angles was studied with high emphasis placed on the temperature change, thermal equilibrium state and solid–liquid interface in droplet during freezing. Exact freezing times were impossible to obtain due to the drastic changes in water thermal properties utilized in the presented methodology. However, a similar trend between contact angle and freezing time was obtained experimentally, which proves that this methodology can be used for benchmark investigation. The utilized solidification model was based on an enthalpy-porosity technique [39]. It does not track the liquid/solid interface explicitly but rather is based on enthalpy balance. An additional scalar parameter called liquid fraction is introduced into the calculation region, which determines the percentage of the cell volume which is in liquid/solid form. This mushy zone is treated as a porous medium with the liquid fraction representing porosity value. From this, an additional term is added into the momentum equation, which enables steering the velocity of the fluid in cells based on the liquid fraction [40]. If the value of this parameter decreases, meaning higher solid content, an added source term begins to dominate other terms in the momentum equation. As the value of the liquid fraction approaches 0, the velocity of fluid is also forced to values close to zero.

Another numerical study of the freezing process included the effect of supercooled water in the droplet on a cold, flat plate [8]. A numerical model considering both the volume expansion during the freezing process and the supercooling effect was established using the solidification/melting model from ANSYS Fluent 14.0 software. Simulations were performed on both hydrophilic and hydrophobic surfaces for supercooled droplets of varied sizes (5–40 μL). The freezing front movement and freezing time were studied with image recognition technology and compared with the experimental results. A high degree of accuracy was observed, which was much higher than for models ignoring the supercooling effect or using the initial droplet profile. The proposed model reduced the deviation of freezing time from about 30% to about 10% for both hydrophilic and hydrophobic surfaces.

An additional study [41] presented a numerical model for supercooled water droplets impacting a flat surface. To study ice adhesion at the droplet scale, a methodology was presented to simulate the impact and solidification of a supercooled water droplet on a cooled substrate. Upon impact, nucleation was assumed to occur instantaneously, and properties of the droplet were chosen to account for the nucleation process. The calibration of the constant parameter A_mush_ was necessary for the simulation of droplet solidification with the enthalpy-porosity technique. The mushy zone constant determines the damping magnitude of the fluid velocity in momentum equation. When the A_mush_ values are higher, the velocity of the solidifying fluid decreases more rapidly. It was shown by matching different numerical cases with corresponding experimental data that even such complex phenomenon can be predicted with an accurate simulation of droplet spread and freezing for several droplet impact conditions.

However, not many numerical studies of droplet freezing have included surface roughness. This is largely due to the significant increase in geometric complexity when attempting to simulate the fine surface details in the micrometer range for much larger droplets [42]. In the study of molten metal droplet impingement [43], the surface roughness was averaged with thermal contact resistance. Another approach was presented in [44], where molecular dynamics with machine learning techniques were utilized to study the effect of surface roughness on ice adhesion. However, those methodologies do not directly simulate the effect of surface roughness and do not provide specific information about possible mechanisms of freezing delay observed in experiments.

In this article, a methodology for the numerical simulation of droplet freezing including a micrometer-texturized pattern was developed. Based on the laser-texturized samples of polyurethane-coated metal substrates studied with freezing delay experiments from previous work [18], their numerical models were generated, and droplet freezing simulation was performed. The obtained simulation results of freezing time were compared to experimental measurements for evaluation of the proposed methodology.

## 2. Materials and Methods

**Figure 1:**
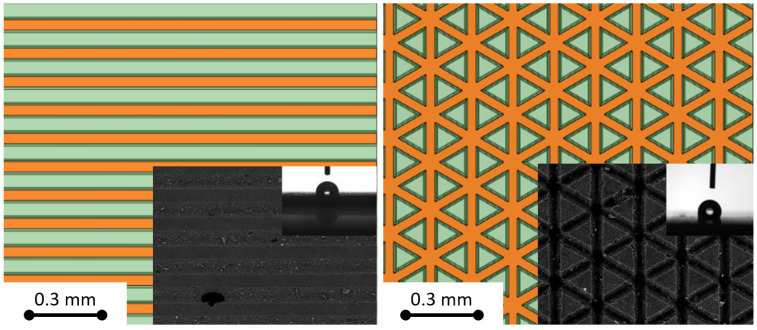
Comparison of studied pattern models with SEM images from Ref. [18] of real texturized surfaces: linear (**left**) and triangular (**right**) pattern. In the images, orange denotes grooves created during laser processing, while green indicates protrusions that were not processed.

The modeling study was based on real material samples from work [18], in which experimental contact angle and freezing delay measurements were performed. Three cases from the above-mentioned paper were studied—non-texturized and texturized with linear and triangular patterns (see Figure 1), all with a 5% addition of the SILOX1 modifier (mixture of vinyltrimethoxysilane and hexene in a molar ratio of 1:2, purchased from Linegal Chemicals, Warsaw, Poland). The wettability of the surfaces was determined by measuring the water contact angles (CA). The measurements were performed using an OCA15 goniometer from DataPhysics Instruments (Filderstadt, Germany) with OCA software (SCA20 6.1, DataPhysics, Filderstadt, Germany) using the sessile drop method. The volume of the water droplet was 5 μL. CA was measured at room temperature (25 °C) after 5 s of stabilization. The CA values are the average of six different measuring points on the surface. The freezing delay time (FDT) of water droplets was determined using an OCA15 goniometer (DataPhysics Instruments, Filderstadt, Germany) with dedicated OCA software, and it was equipped with a chamber designed for testing at reduced temperatures. The test was conducted at −10 °C and lasted a maximum of 4 h. The volume of the water droplets was 5 μL.

**Figure 2:**
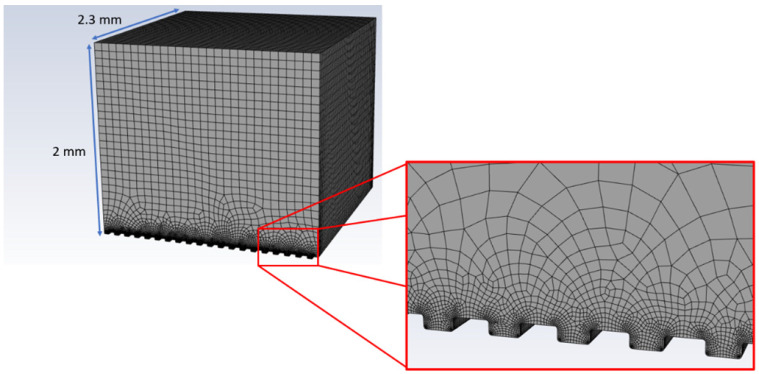
Example of the simulation domain with volume element mesh.

The simulation of a droplet freezing delay experiment was performed using a 3D simulation domain (2.3 mm length, 2.3 mm width and 2 mm height) of area above the material surface, which was generated in Ansys SpaceClaim (ANSYS 19.2, ANSYS Inc., Canonsburg, PA, USA). Micrometric grooves were translated over 20 times to introduce details of roughness generated with laser patterning. Meshing of the simulation domain was performed in an Ansys Design Modeler (ANSYS 19.2, ANSYS Inc., Canonsburg, PA, USA). The global mesh size was set to 75 µm, with 8 µm refinement inside the grooves, which was based on a mesh convergence study for the non-texturized model. This resulted in a total number of cells equal to around 700,000. An example of the meshed simulation domain is presented in Figure 2.

Ansys Fluent (ANSYS 19.2, ANSYS Inc., Canonsburg, PA, USA) was utilized to simulate droplet freezing delay. The procedure was divided into two processes: stabilization and freezing. In the stabilization step, the droplet was dropped onto the material surface and took an equilibrium shape. The volume of fluid (VOF) method was utilized to track the multiphase (water/air) interface. The gravitational forces were included in the simulation. The physical properties of air and water were imported from the ANSYS Fluent 19.2 database. Surface tension was included with standard value of 0.072 N·m^−1^ with the Continuum Surface Force model [45]. Large body forces present in the simulation (surface tension, gravity) required an additional implicit body force correction in the momentum equations. The cell zone operating conditions were set to standard pressure (1013.25 hPa) and the density of the lighter phase (1.225 kg·m^−3^). The wall boundary condition was set from all sides of the simulation domain. On the lower wall representing the material’s surface, the no-slip condition and intrinsic contact angle (101°) were set. No provisions were made for encapsulated air. The air pressure from air between the droplet and surface was assumed to be the same as air pressure in the rest of the simulation, meaning that it was assumed that the air had an escape route.

The Pressure-Implicit with Splitting of Operators (PISO) scheme of pressure-velocity coupling was utilized in the simulation. A very small timestep value of 10^−7^ s was set due to Courant number limitation. The simulation was initialized with no gauge pressure, a volume fraction of 100% air and 0 m·s^−1^ velocity inside the calculation zone. The water droplet was generated as a sphere with a radius of 0.8 mm patched just above the lower surface with a volume fraction of 100% water. The droplet fell onto the lower boundary, representing the material’s surface and the stabilization process was initialized. The first step of the simulation was completed when the average velocity in a whole calculation region reached the minimum value.

After the stabilization procedure, side-view images of the droplet volume fraction were generated in grayscale and exported for apparent contact angle measurement. Due to distortion of the interface between phases, the images were loaded, cropped, and binarized in ImageJ software (version 1.53e). Afterwards, the apparent contact angle was determined using the DropAnalysis plugin, which is based on the B-spline snakes method [46]. To reduce the error related to the distorted interface and manual outlining of the droplet, CA measurements were performed three times for each perpendicular side of the simulation zone.

After droplet stabilization, the second step of simulation was performed—freezing modeling. Energy and solidification/melting modules in Ansys Fluent were enabled from this point. The temperature on the lower boundary was set to −10 °C as a Dirichlet boundary condition. Such simplification was introduced to create a heat sink due to the high thermal conductivity of the aluminum plate cooled with a Peltier cell during the experimental procedure. The rest of the calculation zone was patched with a temperature of 1 °C. Water/ice density correction was adopted with the User-Defined Function (UDF)—the density of solid fraction was set to 0.917 kg·m^−3^. The mushy zone constant was set to 5 × 10^4^ based on the work [41]. Freezing simulation was performed until the whole droplet froze—the liquid fraction value in the cells associated with the droplet achieved a value of 0. In every volume element, the system is assumed to be ice if the temperature is below 273 K and water if it is above 273.1 K. In the range between 273 K and 273.1 K, mechanical and thermodynamic properties (e.g., latent heat of freezing) are assumed to linearly scale from those of ice to those of water.

This part of the model is much more complex than just wetting. In addition to the large number of mesh elements, we need to include additional heat transfer equations at a much larger timescale. Droplet stabilization can be simulated on a timescale of seconds, but freezing can take minutes. Initial simulations revealed that it is not possible to simulate even one case in a reasonable time, especially if we want to include tiny details of the surface topography. To deal with this problem, we adopted a similar strategy to that in work [3]. The authors of this paper proposed a solution to this problem by changing the water properties to speed up the freezing process. The latent freezing heat and specific heat capacity were decreased by 1000 times, so the droplet freezing could be studied. In our work, we adopted a similar strategy. However, this method has a major drawback. We cannot predict the freezing delay time accurately due to changes in water properties, but we can perform benchmark testing to compare which surface pattern delays freezing more.

## 3. Results and Discussion

For the non-texturized case, the value of contact angle was the same in both modeling and experimental studies—101° ± 1. For the linear pattern, a slight deviation was observed between the experimental (129° ± 4) and modeling (125° ± 5) results. The surface with the triangular pattern exhibited lower contact angle values: 112° ± 3 in the experiment and 111° ± 6 in modeling. A much better fit between the experimental and modeling results was obtained than in our previous work [47]. It can be attributed to the change from a 2D to 3D model, which can include the anisotropy of the contact angle depending on the direction of measurement.

**Figure 3:**
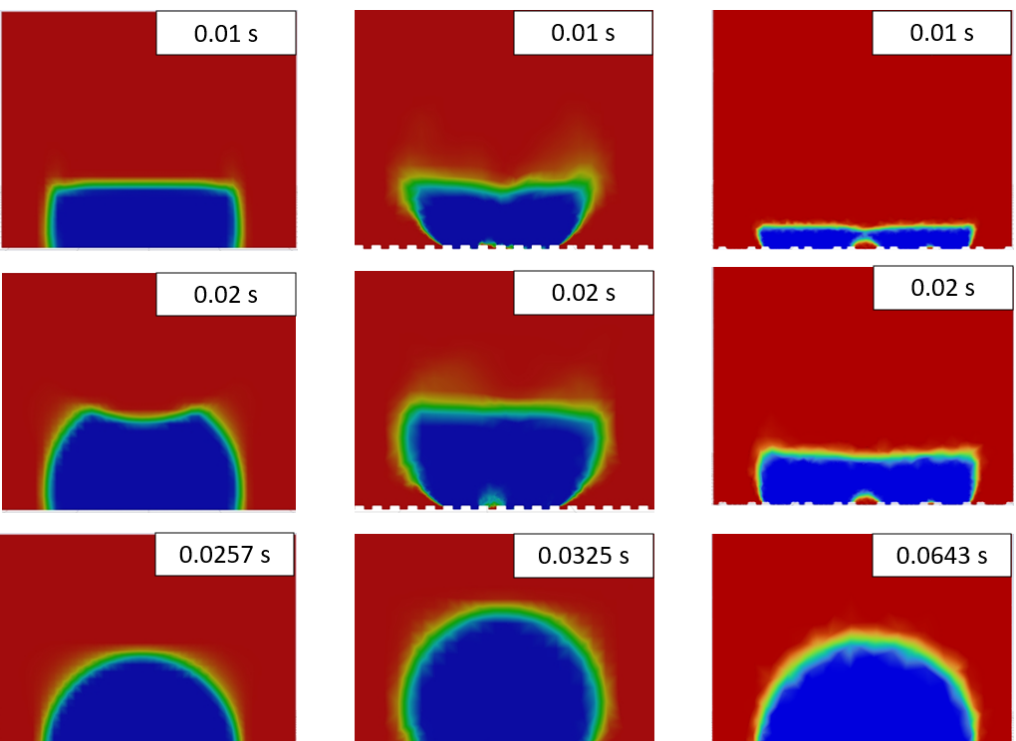
Time snapshots of the water droplet freezing for flat surface (**left**), linear pattern (**middle**) and triangular pattern (**right**). In the images, blue denotes a liquid fraction value of 0 (solid), while red indicates a liquid fraction of 1 (fluid).

The results of droplet freezing modeling are presented in Figure 3. For the non-texturized case, the standard evolution of the ice front during droplet freezing was observed. The process finished after 25.7 ms. Such a small value is resulting from the downscaling of water thermal properties. For texturized surfaces, considerable variations in ice front propagation were observed. However, the shape of the completely frozen droplets obtained after complete freeze was regular. Due to the change in density during the freezing process, the ice has partially filled the grooves of a linear pattern. For the triangular pattern, water partially infiltrated the grooves, creating bubbles underneath the droplet, which were present during the freezing simulation. Additionally, the freezing delay time was higher for the triangular pattern (64.3 ms) compared to the linear pattern (32.5 ms).

In Table 1, a comparison between the modeling and experimental results is presented. The apparent contact angle results for modeling and experiments were similar. Significant changes in water properties caused the results of the freezing time for modeling and experiments to be of different orders of magnitude—milliseconds and minutes. The rescaling of latent freezing heat and specific heat capacity is tantamount to increasing the thermal conductivity by the same factor. Since, in our system, the heat is transported away through the ice layer, the appropriate thermal conductivity is that of ice, while what was used in the simulations was the thermal conductivity of water (four times less than ice), altogether this gives 250 times rescaling of the freezing rate. The remaining discrepancy (a factor of approx. 200) may be attributed to the exclusion of possible additional thermal contact resistance across the ice-substrate interface and the fact that the substrate is modeled as a metal at infinite thermal conductivity, while the experiment was carried out on a polymer surface. A systematic study of computational grid-point density would have been preferrable but is beyond the capability of the available computer resources.

However, approximately the same increase in freezing delay time was observed due to texturization with the linear pattern—25.3% in experiments and 26.5% in simulation. Discrepancy was observed for the triangular pattern—in the experiment, the increase due to texturization was over 420% compared to 250% from the simulation. Hence, the developed methodology cannot accurately predict freezing time, but it can give qualitative trends in freezing delay capabilities for different texturized patterns.

**Figure 4:**
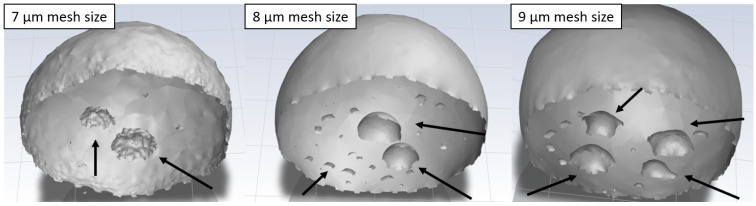
Influence of small mesh variations on the bubble distribution under the droplet on a surface with a triangular pattern: mesh refinement sizes of 7 µm (**left**), 8 µm (**middle**), and 9 µm (**right**). Arrows points to the observed bubbles beneath the droplet.

Due to the discrepancy in the observed results of freezing delay time increase due to texturization for the triangular pattern, additional models with different mesh refinement sizes (7 µm and 9 µm) were generated. This was especially interesting due to the observed mixed wetting state. The study revealed that the formation of bubbles underneath the droplet during the stabilization procedure is highly dependent on the mesh structure. Even for small changes in mesh refinement size, completely different bubble distributions were observed (see Figure 4). It may be caused by the so-called spurious currents, which are commonly found in VOF models with surface tension. The blurry nature of the interface in the VOF models leads to errors in the calculated curvature. The non-physical velocities near the interface are generated, which are dependent on mesh geometry [48]. The different bubble distributions changed the freezing delay time from 61 to 73 ms, depending on the configuration. Furthermore, the less emphasized increase in freezing delay time for the modeling case may be caused by a constant temperature boundary condition, which does not take into account the heat transfer through the material. In the case of water infiltrating the grooves during the stabilization, heat flux in following freezing simulation is more complicated due to the presence of perpendicular wetted facets in the pattern. Hence, this methodology should be improved to study more complicated wetting states.

Additionally, the mixed wetting state and formation of trapped air bubbles under the droplet may be the reason why the contact angle is much smaller for a triangular pattern compared to a linear pattern. During experimental measurements of the contact angle, it was not possible to observe the material/water interface in high detail, so it was unclear if any air bubbles were trapped under the droplet. The proposed model renders it possible to analyze additional aspects of wetting and freezing of the droplet on a rough surface, which may clarify these processes.

## 4. Conclusions

In this work, the methodology of droplet freezing simulation on texturized surfaces was presented. Obtained modeling results of the apparent contact angle and freezing delay time were compared with experimental values. Similar wetting characteristics were observed in all cases. The decrease in latent freezing heat and specific heat capacity of the water phase caused the results of freezing delay time to be of different order of magnitude, which eliminates the possibility of accurately predicting the freezing delay time in the simulation. However, a similar increase in freezing delay was observed, which enables the benchmark testing of icing delay for various surface patterns.

This modeling procedure is a promising alternative, serving as a complementary tool to experimental testing and offering additional insights into air distribution beneath the droplet—insights that are not easily obtainable through experimental techniques. In future work, we plan to extend this study by incorporating a broader range of surface textures and investigating additional parameters, such as ice adhesion strength, to provide a more comprehensive understanding of how roughness affects the icephobic properties of the surface.

## Figures and Tables

**Table 1:** Comparison between modeling and experimental results [18].

Sample	Experimental			Model		
	Apparent Contact Angle [deg]	Freezing Delay Time [s]	Increase Due to Texturization [%]	Apparent Contact Angle [deg]	Freezing Delay Time [s]	Increase Due to Texturization [%]
Non-texturized (flat)	101 ± 3	1134	-	101 ± 1	0.0257	-
Linear pattern	129 ± 4 (115 µm spacing distance)	1422	25.3	125 ± 5 (115 µm spacing distance)	0.0325	26.5
Triangular pattern	112 ± 3 (163 µm spacing distance)	>4800	423.3	111 ± 6 (163 µm spacing distance)	0.0643	250.2

## Data Availability

The original contributions presented in this study are included in the article. Further inquiries can be directed to the corresponding author.
